# Efficacy of a chewable tablet containing sarolaner, moxidectin, and pyrantel (Simparica Trio^®^) in the treatment of sarcoptic mange caused by *Sarcoptes scabiei* mite infestations in dogs

**DOI:** 10.1186/s13071-023-06049-9

**Published:** 2023-11-27

**Authors:** Csilla Becskei, Julian Liebenberg, Tiago Fernandes, Stasia Borowski, Lina D’Hanis, Sean P. Mahabir

**Affiliations:** 1 Zoetis, Veterinary Medicine Research and Development, Mercuriusstraat 20, B-1930 Zaventem, Belgium; 2 ClinVet International (Pty) Ltd, Uitzich Road, Bainsvlei, 9338 Bloemfontein, Republic of South Africa; 3 Zoetis, Veterinary Medicine Research and Development, 333 Portage St, Kalamazoo, MI 49007 USA

**Keywords:** Efficacy, Oral, Sarolaner, Moxidectin, Pyrantel pamoate, Simparica Trio^®^, Dog, Sarcoptic, *Sarcoptes scabiei*, Mange, Scabies

## Abstract

**Background:**

Infestation with *Sarcoptes scabiei* in dogs is a debilitating disease if left untreated and is transmissible to humans. Two field studies were conducted to confirm the efficacy of orally administered sarolaner in combination with moxidectin and pyrantel (Simparica Trio^®^) in the treatment of sarcoptic mange in dogs.

**Methods:**

Client-owned dogs with *S. scabiei* infestation were enrolled and received 2 monthly treatments. In the first, small-scale study, 12 dogs each were allocated randomly to treatment with either placebo or Simparica Trio^®^. Skin scrapings to detect live mites and assessment of clinical signs of sarcoptic mange were conducted on Days 0, 14, 30, 44, and 60. Efficacy was calculated based on the percent reduction in arithmetic mean live mite counts relative to placebo. In the second, large-scale study, 75 dogs were allocated randomly to treatment with Simparica Trio^®^ and 37 to treatment with afoxolaner + milbemycin oxime (NexGard Spectra^®^). Skin scrapings to detect live mites and assessment of clinical signs of sarcoptic mange were conducted on Days 0, 14, 30, and 60. The parasitological cure rate (percentage of dogs without live mites) was determined and non-inferiority of Simparica Trio^®^ to the control product was assessed.

**Results:**

In the small-scale study, 2 monthly doses of Simparica Trio^®^ resulted in a significant reduction (*P* ≤ 0.0050) in live *S.* *scabiei* mite numbers and provided a 99.2% reduction relative to placebo by Day 60. Clinical signs of sarcoptic mange improved throughout the study in Simparica Trio^®^-treated dogs. In the large-scale study, the parasitological cure rate on Days 30 and 60 was 97.3% and 100% in the Simparica Trio^®^ group and 91.9% and 100% in the afoxolaner + milbemycin oxime group, respectively. The parasitological cure rate for Simparica Trio^®^ was non-inferior to afoxolaner + milbemycin oxime at both time points. Clinical signs of sarcoptic mange improved throughout the study in both groups.

**Conclusions:**

Two-monthly doses of Simparica Trio^®^ reduced *S. scabiei* mite counts by 99.2% relative to placebo in one study and eliminated *S. scabiei* mites in 100% of dogs in the second study, thus confirming that Simparica Trio^®^ is highly effective in the treatment of sarcoptic mange in dogs caused by *S. scabiei* var. *canis.*

**Graphical abstract:**

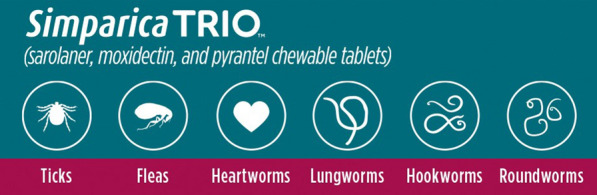

## Background

Sarcoptic mange (also known as scabies) is a highly contagious and intensely pruritic dermatological condition in dogs caused by infestation with *Sarcoptes scabiei* var. *canis*. The mite has a worldwide distribution, and infestations can occur in all domestic dogs and wild canids, especially coyotes, wolves, and the red foxes [1]. Most infestations are acquired by direct contact with an infested dog or fox; however, transmission by indirect contact (e.g. contact with bedding or grooming tools from an infested animal) can also occur [2].

*Sarcoptes scabiei* mites burrow in the lower stratum corneum of the skin, and this results in the clinical signs typically associated with sarcoptic mange, which include intense pruritus, alopecia, and hyperkeratotic lesions that commonly affect the predilection sites of the ear margins, periocular region, face, lateral elbows, and lateral hocks [1]. A positive pinnal-pedal scratch reflex is also present in many dogs [3]. Young or immunocompromised animals may develop “crusted scabies,” which is characterized by marked hyperkeratosis with extremely large numbers of mites, although paradoxically these animals are not usually pruritic [1].

Diagnosis of sarcoptic mange can be confirmed by identifying live *S. scabiei* mites and/or eggs by skin scraping. Finding mites can be difficult, especially in early disease when mite numbers are relatively low; therefore, it is recommended that multiple skin scrapings be made to improve mite detection [4]. Fecal flotation and histopathology of skin biopsies are alternative methods for mite detection [2]. Recently, PCR assay on skin swabs has been reported to be useful in the diagnosis of infestations as well [5]. In cases where sarcoptic mange is strongly suspected but mites cannot be found, a therapeutic drug trial is often instituted and the diagnosis made based on response to treatment.

Most dogs with sarcoptic mange respond well following treatment with an acaricidal agent that is effective against *S. scabiei* mites [1]. Although most widely known for their efficacy against fleas and ticks, the isoxazoline group of ectoparasiticides has also demonstrated excellent efficacy against mites that commonly infest dogs, including *S. scabiei* [6]. One example is sarolaner, which at the 2 mg/kg minimal dose (Simparica^®^) is approved by the European Medicines Agency (EMA) for use in the treatment of sarcoptic mange [7] and has demonstrated 100% efficacy against *S. scabiei* mites following 2 monthly oral doses under laboratory and field conditions [8].

Here, we report on two field studies that were conducted to confirm the efficacy of sarolaner at the 1.2 mg/kg minimal dose level in combination with moxidectin and pyrantel (Simparica Trio^®^) against natural infestations of *S. scabiei* in dogs.

## Methods

Both studies were randomized, double-masked studies, utilized client-owned dogs, and were conducted according to Good Clinical Practice Guidelines [9]. Dogs enrolled in either study had clinical signs of sarcoptic mange, which included pruritus, erythema, scaling, crusting, papules, and hair loss. In addition, live *S. scabiei* mites (immature or adult) were identified on skin scrapings prior to treatment. In the small-scale study, a minimum of five live mites were required, and in the large-scale study at least one mite was required. Dogs were kept in their normal domestic environment. The dogs were fed and watered according to the normal practice of each dog’s owner. If more than one dog in a household met the inclusion criteria, the dog with the most severe clinical signs of sarcoptic mange was selected as the primary patient and that dog was used for efficacy evaluations. Due to the contagious nature of sarcoptic mange, all other dogs in the household had to be enrolled in the study, were considered as “supplementary dogs”, and had to receive the same treatment as the primary dog. Skin scrapings for mite counts and assessment of clinical signs were only performed on primary dogs and therefore only primary dogs were included in the efficacy analysis. In the safety summary all primary and supplementary dogs were included.

Owners and all personnel involved in making assessments of efficacy or safety were masked to treatment assignments. Day 0 was defined as the day of first treatment.

### Small-scale field study

#### Animals

Dogs were enrolled in South Africa. Dogs at least 6 months of age and at least 1.3 kg bodyweight were selected for the study. Both sexes were represented, and females were confirmed not to be pregnant or lactating. All dogs were individually identified by microchip and assessed as being in good health at the time of enrollment based on physical examination by a veterinarian. Dogs treated with an ectoparasiticide that had residual activity against *S. scabiei* mites at the time of enrollment and those with concurrent demodectic mange were excluded from participation.

#### Mite counts

Mite counts were conducted on Days 0, 14, 30, 44, and 60. Mite infestations were evaluated by skin scrapings to an approximately consistent depth using a blunted scalpel blade over an area of approximately 2.5 cm^2^. Scraping continued until capillary bleeding occurred. Scrapings were taken from at least four separate sites on each dog. If no mites were detected in the first four scrapings, then additional scrapings were made until live mites were found or the maximum of 10 scrapings was reached. Selected scraping sites were those that had the most severe or likely evidence of current mite infestation. If no obvious lesions were present, then predilection sites (e.g. ear margins, elbows, hocks, and ventral chest) for harboring mites were scraped. The collected material was transferred to mineral oil on a microscope slide and live *S. scabiei* mites (larvae, nymphs, and adults) and any eggs present were counted using low magnification.

#### Clinical sign assessment

Clinical signs of sarcoptic mange were assessed before skin scraping on each mite count day. Each dog was thoroughly examined for skin lesions characteristic of sarcoptic mange including pruritus, erythema, scaling, crusting, papules, and hair loss. Each dog was observed for approximately 5 min to evaluate pruritus. The severity of each clinical sign was scored as absent (no observable abnormality); mild (intensity/density is low and only a small area is affected); moderate (great intensity/density over a small area or lesser intensity/density but affecting a large area); and severe (great intensity/density and covers a large area). In addition, the body surface area affected by each lesion was recorded on a dog silhouette, and the percentage of total body surface area affected was estimated.

#### Randomization

Primary dogs were allocated to one of the two treatments according to a pre-determined randomized complete block design with a one-way treatment structure. Dogs were blocked based on order of enrollment, and within each block dogs were allocated randomly to treatment with either placebo or Simparica Trio^®^. Any supplementary dogs in the household received the same treatment as the primary dog.

#### Treatment

Treatments were administered to all primary and supplementary dogs on Days 0 and 30. Doses were calculated based on the body weight collected immediately prior to each dosing. According to the label of Simparica Trio^®^, there are no restrictions regarding the prandial state at the time of treatment administration; therefore, tablets could be administered at any time regardless of the dog’s normal feeding schedule.

Dogs allocated to treatment with Simparica Trio^®^ were dosed with single tablets or a combination of tablets of different strengths to receive the minimum dosages of 1.2 mg/kg sarolaner + 24 µg/kg moxidectin + 5 mg/kg pyrantel (as pamoate salt) as closely as possible without underdosing. Dogs allocated to treatment with placebo were dosed according to the same dosing chart and received tablets made with the Simparica Trio^®^ formulation but without the active ingredients.

Treatments were administered by the dispenser by hand pilling to ensure accurate dosing. Each dog was observed for several minutes after dosing for evidence that the dose was swallowed and for potential adverse events associated with forced administration of a whole chewable tablet.

Following conclusion of the study, all dogs received treatment with an approved miticidal agent with efficacy against *S. scabiei* based on the recommendations of the study veterinarian and the consent of the dog owner.

#### Safety assessments

Physical examinations were performed by a veterinarian on all primary and supplementary dogs on Days 0 (prior to treatment), 14, 30, 44, and 60. All abnormal health events observed by the veterinarian during physical examinations or observed by the owner were recorded, as were any concomitantly administered medications. Each dog owner was provided with a diary to record any abnormal health observations that occurred between study visits, and every effort was also made to contact each owner by phone to confirm the health status of enrolled dogs on Days 7, 21, 37, and 51.

#### Data analysis

All dogs that received treatment were included in the safety assessments. Only primary dogs were included in the efficacy analysis. The experimental unit was the primary dog in each household, and the primary endpoint was the live (adult + nymph + larvae) mite counts.

Post-treatment percent reduction in live mite counts relative to placebo based on arithmetic means was calculated as follows:

$$\% \ {\text{reduction}} = 100 \times \frac{{{\text{mean}} \ {\text{count}} ({\text{placebo}}) -{\text{mean}}\ {\text{count}} ({\text{treated}}) }}{{{\text{mean}}\ {\text{count}} ({\text{placebo}})}}$$

Post-treatment percent reduction in live mite counts relative to pre-treatment based on arithmetic means was calculated as follows:

$$\% \ {\text{reduction}} = 100 \times \frac{{{\text{mean}}\ {\text{count}} ({\text{pre-treatment}}) - {\text{mean}}\ {\text{count}} ({\text{post-treatment}}) }}{{{\text{mean}}\ {\text{count (pre-treatment)}}}}$$

Mite counts were natural log transformed [log_e_ (count + 1)] prior to analysis. Transformed counts were analyzed using a mixed linear model for repeated measures. The model included the fixed effects of treatment, day of study, and treatment by day of study interaction. The random effects were block, the interaction between block and treatment (animal term), and error. Treatment differences within day of study and mean differences between pre- and post-treatment time points within treatment were assessed at the two-tailed 5% level of significance.

##### Large-scale field study

#### Animals

Dogs were enrolled from 27 veterinary practices in France (11 sites), Italy (8 sites), and Portugal (8 sites). The patient population was recruited from dogs at least 8 weeks of age and at least 2.0 kg bodyweight. There were no breed or gender restrictions; however, pregnant females or those intended for breeding were not eligible for enrollment. Dogs living in or with a history of travel to heartworm-endemic regions were confirmed to be negative for heartworm (*Dirofilaria immitis*). Enrolled dogs had not received treatment with any ectoparasiticide having residual efficacy against *Sarcoptes* spp., nor had they received any long-acting compounds against *D. immitis* that may have provided efficacy during the study period.

#### Mite counts

Skin scrapings for detection of live *S. scabiei* mites were conducted on Days 0, 14, 30, and 60 using the same procedures as described above for the small-scale study with the exception that mites were not counted, but instead the presence or absence of live larvae, nymph, or adult mites was recorded.

#### Clinical sign assessment

Clinical signs of sarcoptic mange were evaluated using the same procedures as those described above for the small-scale study with the exception that the estimated percent of body surface affected by clinical signs of mange was not collected. Assessments were conducted on all primary dogs on Days 0, 14, 30, and 60 before skin scraping or treatment, where applicable.

#### Randomization

Primary dogs were allocated to one of the two treatments according to a pre-determined generalized, randomized, complete block design with a one-way treatment structure replicated in multiple clinics. At each clinic, dogs were blocked based on order of enrollment in groups of three and allocated randomly to treatment with either Simparica Trio^®^ or NexGard Spectra^®^ (afoxolaner + milbemycin oxime) in a 2:1 ratio. Any supplementary dogs in the household received the same treatment as the primary dog.

#### Treatment

Treatments were administered to all primary and supplementary dogs on Days 0 and 30. Doses were calculated based on the body weight collected immediately prior to each dosing.

Simparica Trio^®^ was provided in six different tablet strengths to provide dose ranges of 1.2–2.4 mg/kg sarolaner, 24–48 µg/kg moxidectin, and 5–10 mg/kg pyrantel (as pamoate salt) according to the commercial label instructions. NexGard^®^ Spectra was dosed according to the commercial product label instructions.

There were no restrictions regarding the prandial state at the time of treatment administration; therefore, tablets could be administered at any time regardless of the dog’s normal feeding schedule. The dispenser at each study site was responsible for treatment administration. There were no restrictions on the method of tablet administration; therefore, tablets could be administered by pilling or offered free choice at the discretion of the dispenser. Each dog was observed for several minutes to ensure the dose was complete.

#### Safety assessments

Physical examinations were performed by a veterinarian on all primary and supplementary dogs prior to treatment administration on Day 0. Post-treatment, physical examinations were performed on all primary dogs on Days 14, 30, and 60. Physical examinations on supplementary dogs followed the same schedule as the primary dog with the exception that the Day 14 physical examination was optional and not required. All abnormal health events observed by the veterinarian during physical examinations or observed by the owner were recorded, as were any concomitantly administered medications.

#### Data analysis

All dogs that received treatment were included in the safety assessments. Only primary dogs were included in the efficacy analysis. The primary dog in each household was the experimental unit, and the primary endpoint was the presence/absence of live *S. scabiei* mites (larvae, nymphs, or adults). Live mites present (Yes/No) was analyzed using StatXact as a generalized mixed linear model was not feasible for the observed data. StatXact software was used to construct one-sided 97.5% exact lower confidence limits for the difference between parasitological cure rates (no live mites present) for each treatment group. Parasitological cure rates were estimated using the percentage of dogs in the given treatment group having no live mites in the skin scrapings on the respective study day.

Non-inferiority of Simparica Trio^®^ to afoxolaner + milbemycin oxime for parasitological cure was assessed at each time point using a 15% non-inferiority margin. In effect, this meant that if the one-sided exact lower confidence limit of the treatment difference (Simarica Trio^®^—afoxolaner + milbemycin oxime) was > 0.15, then Simparica Trio^®^ was declared non-inferior to afoxolaner + milbemycin oxime at that time point.

## Results

### Small-scale study

#### Patient demographics

Twenty-four primary dogs (12 placebo and 12 Simparica Trio^®^) and seven supplementary dogs (3 placebo and 4 Simparica Trio^®^) were enrolled and treated (Table 1). One primary dog in the Simparica Trio^®^ group went missing after Day 44 and therefore was not available for evaluation on Day 60.

**Table 1:** Demographics of primary dogs enrolled in the two field studies

	Small-scale study		Large-scale study	
	Placebo (*n* = 12)	Simparica Trio^®^ (*n* = 12)	Simparica Trio^®^ (*n* = 75)	Afoxolaner + Milbemycin Oxime *(n* = 37)
Purebred	0	0	44	18
Non-purebred	12	12	31	19
Age mean (years)	3.9	5.1	4.2	4.6
Age range (years)	0.8–8.0	0.8–9.0	0.2–13.0	0.3–12.0
Bodyweight mean (kg)	14.1	12.6	18.4	16.3
Bodyweight range (kg)	5.9–26.2	8.6–18.7	2.1–50.4	2.5–39.2
Male	5	9	39	21
Female	7	3	36	16

#### Safety

Observations of abnormal health were typical of the type expected in a population of dogs with sarcoptic mange, and none of the events were considered related to the study treatments. No concomitant medications were administered to any dog during the study.

In the placebo group, mild bilateral ocular discharge was observed in two dogs, single episodes of emesis were reported in two dogs, mild hemorrhage was reported in one dog, and a bite wound was observed in one dog. Lethargy, secondary to severe dermatitis, was diagnosed in one dog. In the Simparica Trio^®^ group, mild ocular discharge was observed in two dogs, and hemorrhage and emesis were reported in one dog.

#### Efficacy

Two monthly doses of Simparica Trio^®^ resulted in a significant reduction (*P* ≤ 0.0050) in *S. scabiei* mite numbers and provided a 99.2% reduction relative to placebo by Day 60 (Table 2). Relative to pre-treatment counts, post-treatment live mite counts in the Simparica Trio^®^ group were also significantly reduced (*P* ≤ 0.0004) with a 99.0% reduction by Day 60. On Day 60, nine of 11 Simparica Trio^®^-treated dogs were cured of mite infestation while 11 of the 12 placebo-treated dogs continued to harbor mite infestations: only a single live mite and no eggs were found on the two Simparica Trio^®^-treated dogs while up to 138 mites and 38 eggs were detected on the placebo-treated dogs.

**Table 2:** *Sarcoptes scabiei* live mite and egg counts in the small-scale field study

	Study day				
	0	14	30	44	60
Placebo					
Number of dogs	12	12	12	12	12
Mite counts					
Mean	41.2	23.5^1^	20.9^1^	22.7^1^	22.2^1^
Range	6–206	2–138	1–130	0–135	0–138
% Reduction relative to pre-treatment	-	42.9	49.2	44.9	46.2
% of mite-free dogs	-	0	0	8.3	8.3
Egg counts					
Mean	8.6	1.0	4.6	2.3	3.9
Range	0–53	0–7	0–40	0–18	0–38
Simparica Trio^®^					
Number of dogs	12	12	12	12	11
Mite counts					
Mean	18.6	3.4^1,2^	5.3^1^	1.6^1,2^	0.2^1,2^
Range	8–40	0–9	0–19	0–13	0–1
% Reduction relative to pre-treatment	-	81.6	71.7	91.5	99.0
% Reduction relative to placebo	54.9	85.5	74.9	93.0	99.2
% of mite free dogs	-	16.7	16.7	58.3	81.8
Egg counts					
Mean	4.5	0.0	0.3	0.1	0.0
Range	0–20	0–0	0–3	0–1	0–0

^1^Post-treatment count significantly lower than pre-treatment (*P* ≤ 0.0004)

^2^Simparica Trio count significantly lower than placebo (*P* ≤ 0.0050)

Clinical signs of sarcoptic mange improved throughout the study in Simparica Trio^®^-treated dogs (Table 3). Prior to the first treatment, lesions were present on a mean of 42% of the body surface area of Simparica Trio^®^-treated dogs, and this had reduced to only 8% by Day 60. In contrast, the percentage of body surface area with lesions in placebo-treated dogs remained unchanged at 53% affected prior to treatment and 52% affected on Day 60. All Simparica Trio^®^- and placebo-treated dogs had pruritus, erythema, scaling, crusting, papules, and hair loss before treatment. Signs were severe for pruritus, erythema, scaling, crusting, papules, and hair loss in 58.3%, 50.0%, 50.0%, 50.0%, 50.0%, and 50.0.5% of Simparica Trio^®^ dogs and in 75.0%, 66.7%, 75.0%, 75.0%, 58.3%, and 66.7% of placebo-treated dogs pre-treatment. At study completion none of the clinical signs were severe in the Simparica Trio^®^-treated group, while severe pruritus, erythema, scaling, crusting, papules, and hair loss were present in 16.7%, 25.0%, 33.3%, 25.0%, 8.3%, and 50.0% of placebo-treated dogs.

**Table 3:** Clinical signs of sarcoptic mange in the small-scale field study

	Study day				
	0	14	30	44	60
Placebo					
Number of dogs	12	12	12	12	12
Percent diseased body surface area	53	63	67	50	52
Pruritus	100	91.7	100	100	91.7
Erythema	100	100	100	100	100
Scaling	100	100	91.7	100	100
Crusting	100	91.7	83.3	100	83.3
Papules	100	91.7	66.7	91.7	66.7
Hair loss	100	100	100	100	100
Simparica Trio^®^					
Number of dogs	12	12	12	12	11
Percent diseased body surface area	42	52	46	28	8
Pruritus	100	100	83.3	58.3	9.1
Erythema	100	100	75.0	58.3	18.2
Scaling	100	100	66.7	66.7	18.2
Crusting	100	91.7	66.7	41.7	18.2
Papules	100	75.0	50.0	41.7	9.1
Hair loss	100	100	100	91.7	54.5

For each lesion type, the percentage of dogs with the respective clinical sign is shown

### Large-scale study

#### Patient demographics

One hundred twelve primary dogs (75 Simparica Trio^®^ and 37 afoxolaner + milbemycin oxime) and 81 supplementary dogs (58 Simparica Trio^®^ and 23 afoxolaner + milbemycin oxime) were enrolled and treated (Table 1). All enrolled dogs completed the study. No dogs were withdrawn or lost to follow-up.

#### Safety

Abnormal health events occurred in only three dogs (two Simparica Trio^®^ and one afoxolaner + milbemycin oxime) during the study. In the Simparica Trio^®^ group, one dog was diagnosed and treated for concurrent atopic dermatitis, and one dog was diagnosed and treated for parvovirus. In the afoxolaner + milbemycin oxime group, one dog was diagnosed with motion sickness after vomiting the tablets during the car ride home. The dog was returned to the clinic and was re-dosed with another full dose. None of these events were considered related to study treatment.

#### Efficacy

One hundred twelve primary patients (75 Simparica Trio^®^ and 37 afoxolaner + milbemycin oxime) completed the study and were included in the efficacy analysis. On Day 30, live mites were present in the skin scrapings of two dogs (2.7%) in the Simparica Trio^®^ group and in three dogs (8.1%) in the afoxolaner + milbemycin oxime group. On Day 60, no live mites were detected on any dog in either of the two groups. Parasitological cure rate was thus 97.3% and 100% in the Simparica Trio^®^ group and 91.9% and 100% in the afoxolaner + milbemycin oxime group on Days 30 and 60, respectively (Table 4). The parasitological cure rate for Simparica Trio^®^ was non-inferior to afoxolaner + milbemycin oxime at both time points at a margin of − 15% (Table 5)

**Table 4:** Percentage of *Sarcoptes scabiei*-free dogs in the large-scale field study

	Study day		
	0	30	60
Simparica Trio^®^			
Total number of dogs	75	75	75
Percentage of mite free dogs	-	97.3^*^	100^*^
Afoxolaner + Milbemycin Oxime			
Total number of dogs	37	37	37
Percentage of mite free dogs	-	91.9^*^	100^*^

^*^Parasitological cure rate of Simparica Trio^®^ determined to be non-inferior to afoxolaner + milbemycin oxime because the one-sided exact lower 97.5% confidence interval (CI) was > − 0.15 on both days. Day 30 CI − 0.030, Day 60 CI − 0.049

**Table 5:** Percentage of dogs with each clinical sign in the large-scale field study

	Study day			
	0	14	30	60
Simparica Trio^®^				
Number of dogs	75	75	73^*^	73^*^
Pruritus	97.3	76.0	50.7	13.7
Erythema	94.7	66.7	31.5	9.6
Scaling	96.0	76.0	28.8	8.2
Crusting	97.3	65.3	24.7	2.7
Papules	89.3	52.0	12.3	1.4
Hair loss	98.7	94.7	78.1	27.4
Afoxolaner + Milbemycin Oxime				
Number of dogs	37	37	37	37
Pruritus	97.3	78.4	54.1	8.1
Erythema	91.9	56.8	37.8	8.1
Scaling	97.3	62.2	27.0	10.8
Crusting	91.9	73.0	32.4	0.0
Papules	83.8	48.6	0.0	0.0
Hair loss	97.3	91.9	62.2	13.5

^*^Clinical sign assessment data excluded from two dogs because of concomitant administration of systemic antifungal and Janus kinase inhibitor in one dog and systemic antibacterial administration in the second dog to treat non-study-related concurrent disease

Clinical signs of sarcoptic mange improved throughout the study in both groups. Pre-treatment, numerically more animals had severe clinical signs in the Simparica Trio^®^ group compared to the afoxolaner + milbemycin oxime group as follows: severe pruritus in 45.3% vs. 35.1%, severe erythema in 25.3% vs. 10.8%, severe scaling in 21.3% vs. 10.8%, severe crusting in 12.0% vs. 8.1%, severe papules in 8.0% vs. 0.0%, and severe hair loss in 20.0% vs. 8.1% of the dogs. Nevertheless, at study completion all remaining clinical signs were mild in both groups.

In the Simparica Trio^®^ group, ≥ 89.3% of dogs had pruritus, erythema, scaling, crusting, papules, or hair loss before the first treatment, and this improved with only 13.7%, 9.6%, 8.2%, 2.7%, 1.4%, and 27.4% of the dogs having the same signs on Day 60. Similarly, in the afoxolaner + milbemycin group, ≥ 83.8% of dogs had pruritus, erythema, scaling, crusting, papules, or hair loss before the first treatment, and only 8.1%, 8.1%, 10.8%, 0.0%, 0.0%, and 13.5% had these same signs on Day 60.

## Discussion

The efficacy observed in the current studies confirms that sarolaner remains highly effective against *S. scabiei* when combined with moxidectin and pyrantel. In previous laboratory and field studies conducted with sarolaner administered at the 2 mg/kg minimal dose (Simparica^®^) in dogs with naturally occurring sarcoptic mange, no live *S. scabiei* mites were found on any of the 99 sarolaner-treated dogs approximately 30 days after the second monthly dose, and clinical signs improved following treatment with sarolaner in all three studies [8, 10].

Self-clearing of *S. scabiei* mite infestations is known to occur and is likely a result of acquired immunity to the mite [1, 8]. To allow for the assessment of potential self-clearing in the placebo-controlled small-scale study, in addition to calculating efficacy by comparing mite counts for the Simparica Trio^®^ group to mite counts for the placebo group, efficacy was also calculated by comparing post-treatment mite counts to pre-treatment counts. These comparisons showed that each of the post-treatment mite counts for the placebo group were significantly lower (*P* ≤ 0.0004) than pre-treatment, indicating that some degree of self-clearing of mites likely occurred during the study. Regardless, this had no impact on the interpretation of the study results given that the percentage reduction in mite counts relative to pre-treatment in the placebo group remained relatively constant as the study progressed (42.9% on Day 14 and 46.2% on Day 60). In contrast, in the Simparica Trio^®^ group, the percentage reduction in mite counts increased as the study progressed (81.6% on Day 14 and 99.0% Day 60). Mite counts for the Simparica Trio^®^ group on these days were significantly lower than those for placebo (*P* ≤ 0.0050). In addition, the data support that *S. scabiei* infestation was ongoing in placebo-treated dogs at the completion of the study on Day 60 as evidenced by up to 138 mites being found on 11 of 12 placebo-treated dogs compared to only single mites being found on two of 11 Simparica Trio^®^-treated dogs and the finding of up to 38 eggs in placebo-treated dogs and no eggs being found in any of the Simparica Trio^®^-treated dogs. The latter indicated that Simparica Trio^®^ also interrupted the life cycle of the mites on the treated dogs. The high efficacy of Simparica Trio has been confirmed in the large-scale field study where 100% parasitological cure was achieved in all dogs within 2 months that was accompanied by rapid resolution of the clinical signs of mange infestations.

The isoxazoline group of ectoparasiticides was primarily commercialized for its efficacy against fleas and ticks; however, they have also demonstrated excellent efficacy against the mites that commonly infest dogs, including *S. scabiei*. Relative to older miticidal treatments, the isoxazoline products provide veterinarians with a treatment option that offers greater ease of application and a wider safety margin [6]. Several of the stand-alone isoxazolines are approved in Europe for the treatment of sarcoptic mange in dogs [7, 11, 12], and more recently isoxazoline combination products that expand their spectrum of efficacy to include the treatment and control of intestinal nematodes and the prevention of heartworm disease (*D. immitis*) have also received approval in Europe [13, 14].

The routine use of isoxazoline combination products such as Simparica Trio^®^ for flea and tick control, treatment of intestinal nematode infestations, and the prevention of heartworm disease also provides for the treatment of sarcoptic mange, and year-round use of these products should also prevent establishment of *S. scabiei* infestations in treated dogs.

## Conclusions

Two monthly doses of Simparica Trio^®^ reduced *S. scabiei* mite counts by 99.2% relative to placebo in a small-scale field study and eliminated *S. scabiei* mites in 100% of dogs in a large-scale field study, thus confirming that Simparica Trio^®^ is highly effective in the treatment of sarcoptic mange in dogs caused by *S. scabiei.*

## Data Availability

Data upon which the conclusions are based have been presented in the article.
